# Approaches to working in high-dimensional data spaces: gene expression microarrays

**DOI:** 10.1038/sj.bjc.6604207

**Published:** 2008-02-19

**Authors:** Y Wang, D J Miller, R Clarke

**Affiliations:** 1 Department of Electrical, Computer, and Biomedical Engineering, Virginia Polytechnic Institute and State University, Arlington, VA 22203, USA; 2 Department of Electrical Engineering, The Pennsylvania State University, University Park, PA 16802, USA; 3 Departments of Physiology & Biophysics, Lombardi Comprehensive Cancer Center, Georgetown University, Washington, DC 20057, USA; 4 Departments of Oncology, Lombardi Comprehensive Cancer Center, Georgetown University, Washington, DC 20057, USA

**Keywords:** microarray, gene expression profiling, high dimensionality, data modelling and analysis

## Abstract

This review provides a focused summary of the implications of high-dimensional data spaces produced by gene expression microarrays for building better models of cancer diagnosis, prognosis, and therapeutics. We identify the unique challenges posed by high dimensionality to highlight methodological problems and discuss recent methods in predictive classification, unsupervised subclass discovery, and marker identification.

Gene expression microarrays provide a wealth of information on gene expression patterns and cancer pathways with potential for (1) cancer diagnosis, prognosis, and prediction of therapeutic responsiveness (Ramaswamy *et al*, 2001; Dupuy and Simon, 2007); (2) discovering new cancer subtypes (Golub *et al*, 1999; Lange *et al*, 2004); and (3) identifying cancer-associated (signalling) molecular markers and their complex interactions (Shedden *et al*, 2003; Ransohoff, 2004). However, achieving these biological/clinical objectives requires comprehensive analysis of microarray gene expression profiles that exist in high-dimensional data spaces, and relies critically on the functional capabilities and accuracy of the relevant analytical techniques (Allison *et al*, 2006). Cancer diagnosis/prognosis and therapeutic responsiveness prediction are all supervised classification/prediction problems (Duda *et al*, 2001). Analysing gene expression patterns representing patients that manifest heterogeneous clinical outcomes to discover cancer subgroups amounts to an unsupervised clustering problem (Duda *et al*, 2001). Identification of cancer-associated markers can be cast either as supervised feature/gene selection or as multiple testing, with thousands of candidate markers and a small subset of true ones (Ransohoff, 2004).

Although these analytical tasks fall neatly within statistical learning and pattern recognition (Jain *et al*, 2000), there is nothing conventional about these tasks for microarray data analysis. Unlike conventional pattern recognition that involves moderately dimensioned data, usually less than 100 features per sample and hundreds to thousands of samples, microarrays often involve over 10 000 features/genes per sample (*n*) with typically at most several hundred clinical samples. A rule of thumb is to have at least 10 training samples per feature dimension (Jain *et al*, 2000), whereas in microarrays this ratio is often closer to 0.01 samples per dimension (Allison *et al*, 2006). High feature dimensionality and paucity of microarray samples pose unique challenges for, and inspire novel developments in, predictive classification, cluster discovery, and marker identification methodologies.

A common subtask is feature selection. For predictive classification, only a subset of discriminatory genes is used to avoid overfitting, where a classifier is known ‘too well’ to fit even irreproducible ‘noisy’ training patterns and, thus, to achieve predictive accuracy that generalises well to unseen/test data. In unsupervised clustering in high dimensions, feature selection is likewise essential for discerning the underlying grouping tendency that may be ‘buried’ in a much lower-dimensional subspace – with many structurally irrelevant features and only a small sample size, clustering algorithms are likely to identify false group structure. Lastly, a separate objective is to identify cancer-associated genes and their joint effects, rather than to simply build a predictive model for the disease.

Although feature selection is integral to each of these analytical tasks, an exhaustive search of all 2^*n*^−1 possible feature subsets is prohibitive for large *n*. Thus, practical feature selection techniques are of necessity heuristic, with an inherent accuracy/complexity tradeoff. Moreover, while multivariate analysis methods based on complex criterion functions may reveal subtle joint marker effects, they are also prone to overfitting (Lai *et al*, 2006). Additionally, high dimensionality compromises the ability to validate marker discovery, which requires accurately measuring true and false discovery rates (Ransohoff, 2005). These issues have prompted the development of a variety of novel statistical methods for estimating (or controlling for) false discoveries (Storey, 2003).

## PREDICTIVE CLASSIFICATION

**Figure 1:**
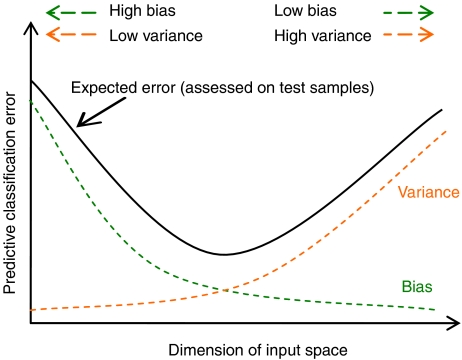
A demonstration of the bias/variance dilemma in predictive classification. Specifically, the error of model fitting can be decomposed into two components, bias (approximation error) and variance (estimation error). Added dimensions can degrade the prediction performance if the sample size is small relative to the dimensionality. For a fixed sample size in the high-dimensional data space, there is a tradeoff between the decreased approximation error and the increased estimation error.

Performance of a predictive model depends on the interrelationship between sample size, data dimensionality, and model complexity. The accuracy of learned models tends to deteriorate in high dimensions, a phenomenon called the ‘curse of dimensionality’ (Duda *et al*, 2001). This phenomenon is illustrated for classification by an example by Trunk (1979). Consider two equally probable, normally distributed classes with common variance in each dimension. For the feature indexed by *n*=1,2,3…, class 1 has mean 1/*n*^1/2^ and class 2 has mean −1/*n*^1/2^. Thus, each additional feature has some class discrimination power, albeit diminishing as *n* increases. Trunk evaluated error rates for the Bayes decision rule, applied as a function of *n*, when the variance is assumed known but the class means are estimated based on a finite data set. Trunk found that (1) the best test error was achieved using a finite number of features; (2) using an infinite number of features, test error degrades to the accuracy of random guessing; and (3) the optimal dimensionality increases with increasing sample size. These observations are consistent with the ‘bias/variance dilemma’ (Jain *et al*, 2000). Simple models may be biased but will have low variance. More complex models have greater representation power (low bias) but overfit to the particular training set (high variance). Thus, the large variance associated with using many features (including those with modest discrimination power) defeats any possible classification benefit derived from these features (Figure 1). With severe limits on available samples in microarray studies, complex models using high-feature dimensions will severely overfit, greatly compromising classification performance. Computational learning theory provides distribution-free bounds on generalisation accuracy in terms of a classifier's capacity, related to model complexity (Vapnik, 1998). Relevance of these bounds to the microarray domain is discussed e.g. by Aliferis *et al* (2006).

There are some strategies for mitigating the aforementioned problem. One is to fit the high-dimensional data, but using simple models that restrict complexity such as naive Bayes models that assume features are conditionally independent or even simpler models that *share* some parameters across classes (Novovicova *et al*, 1996). Another approach is to apply support vector machines (SVMs), which attempt to avoid overfitting by finding a linear discriminant function (or generalised linear discriminant) that maximises the margin (the minimum distance of any sample point to the decision boundary) (Vapnik, 1998). The number of free parameters in SVMs is not a function of the dimensionality, but instead is upper-bounded by the number of samples, which for microarrays is much smaller (Ramaswamy *et al*, 2001). However, whether using linear or nonlinear kernels, SVMs are not immune to the curse of dimensionality. Finally, some methods aim to reduce the amount of parameter learning to avoid overfitting, achieved by regularisation techniques modifying the training objective function or limiting the parameter learning cycles (Duda *et al*, 2001).

Many microarray-based studies suggest that, irrespective of the classification method, feature selection is vital for achieving good generalisation performance (Statnikov *et al*, 2005). The vast number of feature subsets necessitates applying heuristic search techniques, with various accuracy/computation tradeoffs (Guyon and Elisseeff, 2003). Filtering methods apply knowledge of the class labels to evaluate the discrimination power either of individual genes (univariate) or collections of genes (multivariate), based on criteria such as signal-to-noise ratio, correlation measures, and mutual information, before classifier training. A recent study found that for small sample sizes, univariate methods faired comparably to multivariate methods, whose performance may be affected by overfitting (Lai *et al*, 2006).

**Figure 2:**
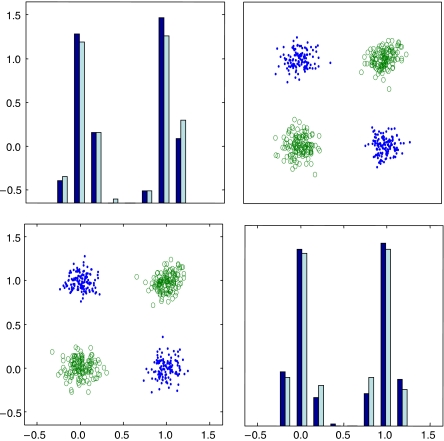
An example of XOR/chessboard-like joint effects. Although the classes consist of disjoint clusters, each variable has completely overlapping class conditional densities, that is, no marginal effect. In contrast, working together, the two variables provide good class separability.

Unlike filtering, wrapper-based approaches combine feature selection and classifier training, with the classifier learning algorithm repeatedly applied for different feature subsets and with the best subset chosen based on a specified criterion (Jain *et al*, 2000). These methods can improve predictive power by capturing higher order (and complex, nonlinear) joint feature effects. Perhaps the simplest example is the ‘noisy XOR problem’, for which two individual features and their linear combinations have no discrimination power, but a simple nonlinear combination is perfectly discriminating (Duda *et al*, 2001; Guyon and Elisseeff, 2003; Figure 2).

Wrapper algorithms, specified by the subset search method and the criterion for evaluating feature subsets, entail large computation in high dimensions, as the number of candidate spaces evaluated grows with the dimension. These algorithms include ‘greedy’ forward selection, with ‘informative’ features added starting from a null set. Other algorithms apply a backward search, which starts from the full space and then eliminates features. Floating (bidirectional) searches, which combine forward and backward steps, and more complex simulated annealing and genetic algorithms, can also be applied (Guyon and Elisseeff, 2003). Finally, there are methods that integrate classifier training and feature selection, such as decision trees, which essentially perform forward feature selection while growing a tree and backward elimination while pruning the tree (Duda *et al*, 2001). For evaluation criteria, either predictive accuracy on held-out test data (Statnikov *et al*, 2005), or criteria that can be evaluated solely on training data such as classifier margin or Bayesian model selection criteria (Guyon *et al*, 2002), can be used.

## UNSUPERVISED CLUSTERING

In microarray data analysis, unsupervised clustering must be cautiously applied and may be unnecessary when samples come with appropriate and reliable supervising labels (Ramaswamy *et al*, 2001; Clarke *et al*, 2008). However, unsupervised clustering constitutes an important tool for discovering underlying cancer subtypes or gene modules (Frey and Dueck, 2007; Miller *et al*, 2008). Such exploration may suggest possible refinement to established cancer categories, where cancer subtypes manifest radically different clinical behaviour and may correspond to distinct biological pathways involving subtype-specific markers (Shedden *et al*, 2003). For example, prostate cancer can be an indolent cancer, remaining dormant throughout life, or an aggressive cancer leading to death. Similar issues arise in drug-resistance cases, where different cancer subtypes exhibit distinctive therapeutic responses (Golub *et al*, 1999).

Furthermore, when therapeutic responsiveness of patients is assessed based on interim growth or shrinkage of a tumour rather than the definitive clinical outcome, unsupervised clustering may be used to validate this supervision information, either to support it or to raise uncertainty about this ‘ground-truth’ if the correlation between the cluster labels and assessed responsiveness is weak. Moreover, trusted class labels on samples can be withheld during unsupervised clustering and subsequently used to validate the clustering methodology/assumptions. Strong correlation between clustering outcomes and known class labels supports the applicability of this clustering approach to other unlabelled microarray data (Golub *et al*, 1999).

While warranted in microarray data exploration, unsupervised clustering is extremely challenging in high dimensions with very few samples. Standard methods such as K-means and hierarchical clustering evaluate distances between data points using all (equally weighted) features. Thus, many noisy/irrelevant features will dominate the (much smaller set of) relevant features in determining how data points are partitioned, for example, many invariantly expressed genes used for microarray normalisation are irrelevant to classification or clustering. Rather than clustering samples using all genes, a practical alternative is to embed gene selection within unsupervised clustering – removal of noisy features improves clustering accuracy, which, in turn, guides a more accurate round of feature selection. Methods have been proposed along these lines (Xing and Karp, 2001; Graham and Miller, 2006), together with novel initialisation schemes (Frey and Dueck, 2007; Wang *et al*, 2007).

**Figure 3:**
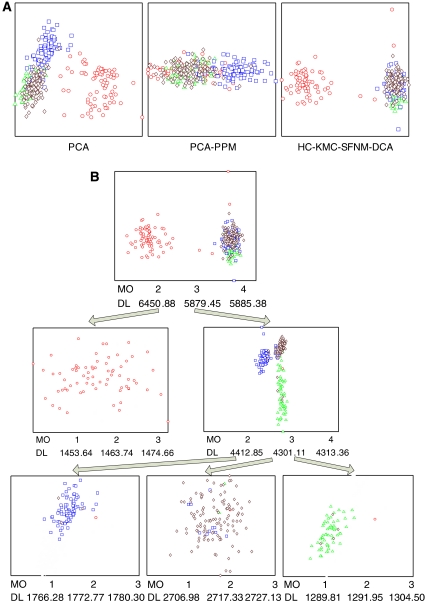
An example of coarse-to-fine top-down divisive unsupervised clustering using VISDA. (**A**) Multiple complementary visualisation subspaces derived from different data structure preserving projection principles. (**B**) Tree of phenotype with embedded model selection function, where MO refers to the model order (number of clusters) and DL refers to the description length (model complexity as a function of cluster number).

Another major challenge for clustering in high dimensions is estimating the number of clusters. Standard methods choose cluster number by best fitting the data while incurring least model complexity. However, under the widely used Bayesian information criterion (Duda *et al*, 2001), model complexity is linear in the number of parameters and quickly grows with each added feature. As many of these parameters model noisy/irrelevant features, their data fitting benefit is grossly outweighed by their contribution to model complexity, which leads to gross underestimation of the number of clusters. In a study by Graham and Miller (2006), a ‘parsimonious’ mixture model allows clusters to share distributions for noisy features, which enhances accuracy in estimating both the cluster parameters and the cluster number in high dimensions. Intrinsic to this modelling is identification of a distinct relevant feature subset specific to each sample cluster, that is, for the microarray domain, each subclass will have its own gene set, as has been conjectured by Shedden *et al* (2003); Ein-Dor *et al* (2005). Another strategy for identifying this cluster structure is top-down divisive clustering that explores and generates hierarchical mixtures in nested subspaces (Wang *et al*, 2007). By projecting high-dimensional data of a current cluster to multiple two-dimensional visualisation subspaces, the human gift for pattern recognition can be exploited to assess the current solution and assist further clustering refinement (Figure 3). Being more data-adaptive and process-transparent, human interaction may bring subjectivity, and thus must be carefully applied.

## MARKER IDENTIFICATION

Marker identification aims to discover those genes and their complex interaction effects that have statistically significant correlations with cancer phenotypes. As it is currently largely unclear how molecular variants and their interactions determine cancer pathogenesis and propensity, marker identification is valuable for improving understanding of the molecular mechanisms of cancers and for suggesting novel drug targets. Discovered markers may also define a subset of networked causal genes that regulate disease phenotype. A review of the current state of this effort is discussed by Aliferis *et al* (2006).

The objectives of feature selection for predictive classification and marker identification bear close resemblance. Although it is tempting to view these two problems as ‘one and the same’, this is often inappropriate. Inclusion of some true cancer markers in a feature set for cancer classification may provide negligible improvement in classification accuracy even though these markers are significantly associated with the cancer outcome of interest. A trivial example is where two markers are perfectly correlated, in which case only one of the two needs to be included in a predictive feature subset. A more interesting example is the one in which, even though two markers are only partially correlated, a classification model will not perceive any benefit from using both markers. This is illustrated below:

Let *A* and *B* take on one of four possible discrete values and suppose the ground-truth statistics on class label *C* are *Prob*[*C*=‘cancer’∣*A*=1]=1.0; *Prob*[*C*=‘cancer’∣*A*=*i*]=0.5, *i*=2,3,4; *Prob*[*C*=‘cancer’∣*B*=3]=1.0; and *Prob*[*C*=‘cancer’∣*B*=*j*]=0.5, *j*=1,2,4. Suppose *Prob*[*A*=1]=0.1; *Prob*[*B*=3]=0.7; and *Prob*[*B*=3∣*A*=1]=0.5. Thus, *A* and *B* are both informative about the disease (for one value), and these variables are only partially correlated. However, in a small training set, it is quite possible that each time *A*=1, *B*=3 also occurs, even though *Prob*[*B*=3∣*A*=1] is much less than one. In this case, while association-based marker discovery might include both *A* and *B*, classification-based marker discovery would only include *B*, because the training set suggests no predictive benefit from including *A*.

More generally, whether predictive gene selection will include a gene that possesses some predictive benefit will depend on the sensitivity of the criterion function used. For example, a predictive model may achieve the same estimated classification error rate using several different feature subsets, even if there is a unique true marker subset, with greatest class discrimination power. Another limitation of predictive gene selection is that most classification models lack interpretability, that is, they do not allow easy discernment of the underlying interactions between the identified markers. The sole focus of most predictive feature selection techniques is on defeating the curse of dimensionality. Exceptions to this include decision trees (if not too large) and Bayesian networks (Duda *et al*, 2001).

Although association-based approaches may ultimately be found superior for identifying cancer markers and their interactions, these methods also have limitations. First, identifying marker interactions, particularly those involving markers with insignificant marginal effect, requires an exhaustive search over the full gene expression space. It is only practical to examine very low-order interactions, for example, ‘10 000 choose 2 or 3′ possible interactions (Jain *et al*, 2000). Thus, higher-order interactions may get missed. One possible strategy is to first apply classification-based gene selection to significantly reduce the search space, followed by (exhaustive search) association-based marker identification. Second, it is difficult to evaluate and/or control inference accuracy for such testing, which involves numerous hypotheses. There is an inherent trade off between statistical power (true positive) and Type 1 error (false positive). Multiple testing for thousands of interacting genes at typical confidence levels leads to unacceptably large false positives. Family-wise error rate techniques can compensate, but conservatively toward minimising false positives and may have insufficient power. Other strategies improve inference accuracy through variance shrinkage that accounts for statistical dependencies between genes via computationally intensive permutation testing to accurately specify the null distribution.

To assess the true statistical significance of the implicated gene subset in multiple testing, one recent method is the randomisation–permutation test (Efron and Tibshirani, 2007). This method addresses the concern that a randomly selected gene subset may appear to possess significant association with the phenotype if only subjected to subject permutation testing. To assure that false discoveries do not occur, a selected gene subset must, additionally, be subjected to a gene randomisation test, where the subject permutation test is to assess whether the implicated gene subset indeed has significant prediction power rather than ‘by-chance’ and the gene randomisation test assesses whether the implicated gene subset has significant prediction power as compared with that of any randomly selected gene subset of the same size.

An additional concern in marker identification is the impact of confounding variables (Ransohoff, 2005). A given data set may represent a biased sample with respect to factors such as patient age, gender, life style or with respect to sample handling, and expression levels for a putative marker may be more strongly associated with these confounding effects than with disease presence (Clarke *et al*, 2008). Although some confounding effects can be mitigated by careful study design or by explicitly accounting for these factors when performing marker identification, further research is needed to devise more effective methodologies for this purpose. Nevertheless, risk factors are not confounding effects to be discounted – there may be cancer-related gene–environment interactions that need to be identified. Finally, there are latent confounding sources due to biological multimodality. For complex phenotypes such as cancers, the presence of multiple, interrelated biological processes may obscure the true relationships between a gene subset and a specific outcome, creating spurious associations that appear statistically correct and yet may be false.

## OUTCOME VALIDATION

In assessing the performance for any of the three fundamental tasks discussed here, a validation procedure must be carefully designed, recognising limits on the accuracy of estimated performance, in particular for small sample size. In the study by Dupuy and Simon (2007), it was shown that, in more than 50% of a representative sample of past studies, inadequate statistical validation was performed. Clearly, classification accuracy must be assessed on labelled samples ‘unseen’ during training. However, single batch held-out test data are often precluded in microarray studies, as there will be insufficient samples for both accurate classifier training and accurate validation. The alternative is a sound cross-validation procedure, wherein all the data are used for both training and testing, but with held-out samples in a testing fold not used for any phase of classifier training, including feature selection and classifier design. Furthermore, performance (for either predictive classification or marker identification) depends on the threshold used to discriminate between categories. Most reported prediction accuracy rates are based on user-defined thresholds for a single operating point. A more meaningful estimate is the receiver operating characteristic curve obtained by using sensitivity (true positive rate) and specificity (true negative rate) acquired at a set of threshold values. The area under the curve gives a comprehensive figure-of-merit for prediction accuracy and can be shown to be a consistent but more sensitive measure than error rate for comparing classifiers, identifying performance differences between classifiers in cases where, evaluated solely by error rate, two classifiers would be deemed equivalent (Swets, 1988; Wang *et al*, 2006).

Unlike predictive classification assessment using labelled samples, validating unsupervised clustering requires alternative avenues when labels are not available. Synthetic data with constructed ground-truth may be used to assess the accuracy of a clustering or cluster number estimation algorithm. However, this approach will not validate that particular statistical assumptions are suitable for fitting molecular profiles from a given population. Alternatively, some form of cross-validation may be used to assess the ‘stability’ of clustering solutions (Lange *et al*, 2004). Stability analysis has been applied to clustering microarrays by Yeung *et al* (2001). Even when class labels are known, Dupuy and Simon (2007) suggest not to use them to select the gene space, as this will bias the clustering results.

It is even less likely to have ground-truth for validating marker identification. Synthetic data constructed from real microarray data can be used to assess a marker identification methodology, with class labels, markers, and interaction models handpicked and treated as ground truth. Importantly, ‘reproducibility’ of marker identification outcomes over multiple/bootstrap data sets may provide reasonable confidence (uncertainty assessment) on the discovered markers (Ransohoff, 2004).

Ultimately, discovered cancer markers or subtypes must be validated against definitive biomedical ground-truth. However, the cost of such validation demands a high degree of confidence in the knowledge extracted from microarray data by marker identification and clustering algorithms. Specifically, such knowledge extraction should not strongly depend on the particular random sample of data used or on variable aspects of the algorithms. Many clustering algorithms find only locally optimal solutions whose quality depends on the pseudorandomly chosen initial cluster parameter values (Frey and Dueck, 2007). Also, greedy sequential feature selection techniques are often ‘unstable’, giving results that may be highly dependent upon the particular training data used. There are two implications. First, whether synthetic data or real microarray data are used, extracted knowledge should be validated by assessing its reproducibility over multiple independently acquired data sets. Independent data sets are easily produced in the synthetic case, but at high cost in the case of real data. The second implication is that algorithms should be made as stable as possible to maximise the generalisation of their results. For marker discovery, one such strategy is to perform marker ranking multiple times, using bootstrap samples and/or *k*-fold cross-validation from the same data set, with the final, selected markers the ones with highest *average* ranking (and perhaps low variance/uncertainty). Nevertheless, the cost of increased stability in such approaches is an increase in computation.
